# Exploratory focused pharmacogenetic testing reveals novel markers associated with risperidone pharmacokinetics in Saudi children with autism

**DOI:** 10.3389/fphar.2024.1356763

**Published:** 2024-02-05

**Authors:** Sireen Abdul Rahim Shilbayeh, Iman Sharaf Adeen, Ezzeldeen Hasan Ghanem, Haya Aljurayb, Khawlah Essa Aldilaijan, Fatimah AlDosari, Abeer Fadda

**Affiliations:** ^1^ Department of Pharmacy Practice, College of Pharmacy, Princess Nourah bint Abdulrahman University, Riyadh, Saudi Arabia; ^2^ Department of Pediatric Behavior and Development and Adolescent Medicine, King Fahad Medical City, Riyadh, Saudi Arabia; ^3^ Pharmaceutical Analysis Section, King Abdullah International Medical Research Center (KAIMRC), King Abdulaziz Medical City, Ministry of National Guard - Health Affairs, Riyadh, Saudi Arabia; ^4^ Molecular Pathology Laboratory, Pathology and Clinical Laboratory Medicine Administration, King Fahad Medical City, Riyadh, Saudi Arabia; ^5^ Health Sciences Research Center, King Abdullah Bin Abdulaziz University Hospital, Princess Nourah bint Abdulrahman University, Riyadh, Saudi Arabia; ^6^ Pharmaceutical Care Department, Ministry of National Guard-Health Affairs, Jeddah, Saudi Arabia; ^7^ Independent researcher, Malaga, Spain

**Keywords:** exploratory, pharmacogenetic testing, autism, risperidone pharmacokinetics, array genotyping

## Abstract

**Background:** Autism spectrum disorders (ASDs) encompass a broad range of phenotypes characterized by diverse neurological alterations. Genomic studies have revealed considerable overlap between the molecular mechanisms implicated in the etiology of ASD and genes involved in the pharmacokinetic (PK) and pharmacodynamic (PD) pathways of antipsychotic drugs employed in ASD management. Given the conflicting data originating from candidate PK or PD gene association studies in diverse ethnogeographic ASD populations, dosage individualization based on “actionable” pharmacogenetic (PGx) markers has limited application in clinical practice. Additionally, off-label use of different antipsychotics is an ongoing practice, which is justified given the shortage of approved cures, despite the lack of satisfactory evidence for its safety according to precision medicine. This exploratory study aimed to identify PGx markers predictive of risperidone (RIS) exposure in autistic Saudi children.

**Methods:** This prospective cohort study enrolled 89 Saudi children with ASD treated with RIS-based antipsychotic therapy. Plasma levels of RIS and 9-OH-RIS were measured using a liquid chromatography–tandem mass spectrometry system. To enable focused exploratory testing, genotyping was performed with the Axiom PharmacoFocus Array, which included a collection of probe sets targeting PK/PD genes. A total of 720 PGx markers were included in the association analysis.

**Results:** A total of 27 PGx variants were found to have a prominent impact on various RIS PK parameters; most were not located within the genes involved in the classical RIS PK pathway. Specifically, 8 markers in 7 genes were identified as the PGx markers with the strongest impact on RIS levels (*p* < 0.01). Four PGx variants in 3 genes were strongly associated with 9-OH-RIS levels, while 5 markers in 5 different genes explained the interindividual variability in the total active moiety. Notably, 6 *CYP2D6* variants exhibited strong linkage disequilibrium; however, they significantly influenced only the metabolic ratio and had no considerable effects on the individual estimates of RIS, 9-OH-RIS, or the total active moiety. After correction for multiple testing, rs78998153 in *UGT2B17* (which is highly expressed in the brain) remained the most significant PGx marker positively adjusting the metabolic ratio. For the first time, certain human leukocyte antigen (HLA) markers were found to enhance various RIS exposure parameters, which reinforces the gut–brain axis theory of ASD etiology and its suggested inflammatory impacts on drug bioavailability through modulation of the brain, gastrointestinal tract and/or hepatic expression of metabolizing enzymes and transporters.

**Conclusion:** Our hypothesis-generating approach identified a broad spectrum of PGx markers that interactively influence RIS exposure in ASD children, which indicated the need for further validation in population PK modeling studies to define polygenic scores for antipsychotic efficacy and safety, which could facilitate personalized therapeutic decision-making in this complex neurodevelopmental condition.

## 1 Introduction

Autism spectrum disorder (ASD) is a complex neurodevelopmental disorder characterized by early onset in youth. However, its exact etiology, involving genetic and nongenetic (i.e., environmental) factors acting either alone or in combination, is still not clear. With the evolution in genomic technology and bioinformatics analysis techniques, several genetic mutations at both the gene and chromosome levels have been identified to be associated with different ASD phenotypes (Genovese and Butler, 2023). Recent reviews of ASD genomics using genome-wide association studies (GWASs) revealed considerable overlap between the molecular mechanisms implicated in the etiology of ASD and certain common genes involved in drug absorption, distribution, metabolism, and excretion (ADME) (Khanzada et al., 2017; Sundararajan et al., 2018; Fang et al., 2023). For example, 11 genes (*SLC6A3*, *UGT*, *GSK3B*, *HTR2A*, *MAOA*, *NOS1AP*, *PDE4B*, *TPH2*, *CACNA1C*, *CHRNA7* and *DRD2*) that influence serotonin and dopamine homeostasis and signal transduction pathways affecting mood, behavior and physical activity in ASD are also known to be associated with the pharmacokinetic (PK) and pharmacodynamic (PD) pathways of drugs employed in ASD management (Butler et al., 2016; Sundararajan et al., 2018; Sheng et al., 2021).

Therefore, it may be possible to observe variations in the PK and PD parameters of drugs in specific ASD patients when compared to other disease populations or normal volunteers (Genovese and Butler, 2020).

As a spectrum disorder, individuals with ASD usually exhibit variable degrees of behavioral and psychiatric manifestations, reflecting heterogeneity in the underlying etiology, which results in the segregation into different ASD phenotypes. This fact highlights the need for the implementation of precision medicine to enable individualization of psychopharmacological drug therapy regimens based on ASD subtype manifestations (Genovese and Butler, 2020). According to recent updates, the medications commonly used to address the comorbidities associated with ASD are atypical antipsychotics, which are frequently employed on a chronic basis according to standard dosing guidelines, which might not fit all etiological subtypes (Aishworiya et al., 2022; Ooi et al., 2023). Additionally, off-label antipsychotic use is still an ongoing clinical practice, despite the lack of evidence for its safety and tolerability (Højlund et al., 2021; Wang et al., 2021; Carthy et al., 2023). This practice was thought to be justified given the shortage of approved clinical cures and was even found to be motivated by advancements in diagnostic and clinician recognition of disparity in ASD and cooccurring mental health issues (Gupta and Gupta, 2023).

Risperidone (RIS) is a U.S. Food and Drug Administration (FDA)-approved atypical antipsychotic medication to target irritability often associated with autistic children (Lamy and Erickson, 2018). However, interindividual variability in RIS effectiveness and safety profiles has been reported in adults and children, even patients with similar diagnoses of psychiatric disorders, including ASD (Lamy and Erickson, 2018; Taurines et al., 2022; Liang et al., 2023). Moreover, RIS PK (exposure) parameters demonstrated wide interindividual variability within children with ASD (Dodsworth et al., 2018; Maruf et al., 2021).

RIS is mainly metabolized in the liver by the CYP450 isoenzyme CYP2D6; however, CYP3A4 and CYP3A5 have also been reported to be partially involved in the 9-hydroxylation of RIS (Fang et al., 1999). 9-OH-RIS is a pharmacologically active metabolite that is approximately equipotent to the parent drug; therefore, both concentrations are collectively referred to as the total active moiety. 9-OH-RIS was later approved by itself as the antipsychotic paliperidone (Clarke et al., 2013). RIS and 9-OH-RIS efflux from cells are affected by certain transporter proteins, such as adenosine triphosphate-binding cassette subfamily B member 1 (ABCB1) (Yasui-Furukori et al., 2004; Saiz-Rodríguez et al., 2018).

Since the transformation of RIS to 9-OH-RIS is mainly mediated by CYP2D6, the ratio of the two molecules (RIS/9-OH-RIS ratio) in blood was classically suggested to be proportional to the CYP2D6 metabolic phenotype (Huang et al., 1993; Cho and Lee, 2006). Therefore, it is assumed that normal healthy subjects with a poor metabolizer (PM) status will have a higher metabolic ratio (less metabolic conversion of RIS) than extensive metabolizers (EMs; usually designated as normal metabolizers [NMs]) and ultrarapid metabolizers (UMs), as both conditions will result in a greater quantity of 9-OH-RIS (Huang et al., 1993; Cho and Lee, 2006; Novalbos et al., 2010). However, a large-scale study involving psychiatric patients of various ages revealed that the positive predictive value of an RIS/9-OH-RIS ratio >1 to predict *CYP2D6* PMs or <1 to predict *CYP2D6* UMs (95% CI) was 35% (26%–46%) and 9% (5%–14%), respectively (Mannheimer et al., 2016). Another pharmacogenetic clinical trial in healthy subjects demonstrated that CYP2D6 predicted only 65% of RIS metabolism variability and highlighted the demand for exploring pharmacogenetic predictors considering the complexity of its PK and PD pathway relationships (Gassó et al., 2014).

Collectively, these results indicated the presence of a potential research scope for examining other genetic markers of non-CYP2D6 variants (such as single-nucleotide polymorphisms (SNPs) involved in genes encoding transporters) (Yoo et al., 2011), which may affect RIS ADME and more comprehensively predict the extent of RIS and OH-RIS exposure (plasma levels) in healthy (Yoo et al., 2011) or unhealthy subjects, such as children with ASD (Troost et al., 2007; Sherwin et al., 2012; Roke et al., 2013; Youngster et al., 2014; Medhasi et al., 2016; Vanwong et al., 2016, 2017; Nuntamool et al., 2017; Rafaniello et al., 2017; Hongkaew et al., 2021; Kloosterboer et al., 2021).

Within the context of ASD, a systematic review of the current state of knowledge regarding CYP2D6 genetic variation and its impact on RIS PK and the propensity for adverse drug reactions in children and adolescents has suggested that CYP2D6 metabolic status was not consistently the sole genetic factor explaining the variabilities within these age groups (Dodsworth et al., 2018; Maruf et al., 2021). Despite the observed trend for a positive association between higher CYP2D6 activity and lower RIS concentration and RIS/9-OH-RIS ratios in some of the included studies (Troost et al., 2007; Youngster et al., 2014; Vanwong et al., 2016, 2017; Nuntamool et al., 2017), there was a consistent nonsignificant difference in the total active moiety concentrations among the different CYP2D6 phenotypes (PMs, EMs, and UMs) in all studies (Troost et al., 2007; Youngster et al., 2014; Vanwong et al., 2016; Nuntamool et al., 2017; Rafaniello et al., 2017; Vanwong et al., 2017). Additionally, these studies were conducted in ASD children of either Caucasian (Troost et al., 2007; Sherwin et al., 2012; Roke et al., 2013; Youngster et al., 2014; Rafaniello et al., 2017) or South Asian (Vanwong et al., 2016; Nuntamool et al., 2017; Vanwong et al., 2017) backgrounds, which could limit extrapolation of the results to other ethnogeographic groups (for example, Saudi Arabians) (McLellan et al., 1997; Al-Dosari et al., 2013) that may carry rare variants that are relatively less frequent in Europeans or South Asians.

Additionally, the few previous studies (Nuntamool et al., 2017; Rafaniello et al., 2017) that attempted to examine a limited number of other typical candidate pharmacogenetic (PGx) markers in RIS ADME (*ABCB1*, *ABCG2*, *CYP3A4*, *DRD2*, *DRD3*, and *HTR2A*) did not have a large enough sample size to make robust conclusions about a panel of genes or their variants or SNPs that should be included for individualized RIS therapy in ASD patients. Given the currently available conflicting data originating from candidate gene association study methods, “actionable” PGx markers related to antipsychotic dosing and selection, in general and with respect to different psychiatric conditions, have a limited application in routine clinical practice, even in developing countries, due to imperfect guidelines for interpretation and implementation (Eap, 2016; Eum et al., 2022).

Based on background information and consistent with the evolution of advanced technology, various pharmacogenomic approaches (targeted, focused or exploratory) can often be employed in human subjects to characterize the genetic determinants that may play roles in various aspects of drug activity and to support ongoing efforts to identify biomarkers predictive of drug exposure and/or safety in specific subgroups of diseases or certain age categories (Burczynski, 2009). Any of the three strategies can be pursued depending on the scale or number of genes in other ADME pathways that need to be examined in parallel (Table 1 in Burczynski, 2009). In our study, as dozens to thousands of genes are suspected to be involved in the drug metabolism or transport of RIS, both exploratory (nonhypothesis) and focused (guided) approaches were justified for identifying genetic alterations in all ADME genes (known PGx markers). Research with a multiplex genotyping approach is relatively innovative and is assumed to provide evidence for better optimum guidance of RIS dosing in physiologically and genetically modified settings, such as children with ASD, to avoid the risk of adverse drug reactions and/or suboptimal responses, as reported in several previous investigations (Correia et al., 2009; Nuntamool et al., 2017; Oshikoya et al., 2019; Shilbayeh S. A. R. et al., 2023).

Given the continuous growth in the knowledge base of DNA polymorphisms associated with ASD risk and ADME of antipsychotics, the current exploratory pharmacogenetic study was conducted with the aim of investigating potential PGx markers involved in RIS exposure (PK) in Saudi children with ASD and achieving a better understanding and clearer insights into the underlying mechanisms of disease-drug-gene interactions in this setting.

## 2 Materials and methods

### 2.1 Study design and participants

This study was a prospective cohort study conducted from November 2020 to February 2021 at three autism centers in Riyadh, Saudi Arabia. All the methodological details, including screening, inclusion of candidate children, and data collection, were described previously in full detail (Shilbayeh S. A. R. et al., 2023).

Blood samples were collected for genotyping and RIS plasma level measurement as described previously (Shilbayeh S. A. R. et al., 2023).

### 2.2 Assay of plasma drug levels

RIS and 9-OH-RIS were extracted and measured in serum samples using a liquid chromatography tandem mass spectrometry system (LC/MS/MS, Waters, USA) according to a previously developed and validated method (Aravagiri and Marder, 2000). The lower limit of quantification of RIS and 9-OH-RIS was 1 ng/mL, and the lower limit of detection for serum RIS was 0.08 ng/mL and for serum 9-OH-RIS was 0.26 ng/mL.

### 2.3 Pharmacogenetic analysis

#### 2.3.1 DNA extraction

Genomic DNA was extracted using the QIAsymphony^SP^ automated extraction system and a QIAsymphony^®^ DSP DNA Midi Kit according to the manufacturer’s instructions (Qiagen, Hilden, Germany). A Nanodrop spectrophotometer (Thermo Fisher Scientific, Santa Clara, CA, USA) was used to determine the concentration and purity of the extracted DNA.

#### 2.3.2 Axiom PharmacoFocus Array

The Axiom PharmacoFocus Array (Catalog identifier: 952396; Thermo Fisher Scientific) offers comprehensive coverage of more than 2,000 markers (SNPs and insertions and deletions) in 150 genes across diverse populations (Tilleman et al., 2019) and functional variants that influence the ADME of commonly prescribed medications that are curated by the Pharmacogenomics Knowledge Base (PharmGKB) with clinical annotation levels of evidence 1A–2B (Whirl-Carrillo et al., 2021). These variants are commonly termed actionable PGx markers for testing in clinical practice (Whirl-Carrillo et al., 2021). Specifically, the Axiom PharmacoFocus Array facilitates genotyping in regions of high homology of key pharmacogenes (*CYP2A6*, *CYP2D6*, *GSTM1*, *GSTT1*, *UGT2B17*, and *SULT1A1*), which are usually difficult to obtain by complex multistep traditional methods (Tilleman et al., 2019).

According to the manufacturer’s instructions, genomic DNA was amplified by multiplex polymerase chain reaction (PCR) using a QIAGEN Multiplex PCR Kit (Qiagen). These amplified products were then fragmented, pooled, resuspended, and hybridized to the PharmacoFocus Array platform (Thermo Fisher Scientific, Santa Clara, CA, USA). The array was scanned on the automated Applied Biosystems™ GeneTitan™ Multi-Channel (MC) Instrument (Affymetrix Inc., Santa Clara, CA, USA). The genotyping call rates and quality parameters of all available markers and samples were generated using Applied Biosystems Axiom™ Analysis Suite software (version 5.2, Thermo Fisher Scientific, Santa Clara, CA, USA).

To achieve the highest genotyping performance, samples that did not satisfy the Dish QC (DQC) parameters were excluded. Furthermore, individuals with a <90% genotyping call rate were excluded from the analyses. Moreover, all markers with any of the following criteria were not considered for the bioinformatic analyses: genotyping call rate <95%, minor allele frequency (MAF) < 0.05, Hardy–Weinberg equilibrium (HWE) *p* < 0.001, and located on the X chromosome.

### 2.4 Statistical and bioinformatic analyses

The identity by descent (IBD) test was performed using PLINK v1.9 (Purcell et al., 2007) to exclude samples with hidden relatedness. To calculate principal components (PCs), we used a total of 8319 probeset markers examined via the PharmacoFocus Array. Subsequently, variants were filtered according to the standard specified criteria: biallelic, passed aligner’s QC, MAF >0.05, HWE *p* > 0.001, and no evidence of linkage disequilibrium (LD) (r^2^ < 0.2). The remaining 4000 variants were used to perform PC analysis with PLINK v2.0 (Chang et al., 2015). Since 84% of the variation was explained by the first two PCs, they were used to remove ancestry and hidden relatedness biases in the association analysis (Supplementary Figure S1).

Association analysis was carried out with PLINK v2.0 (Chang et al., 2015). The 720 selected PGx markers were fitted into a generalized linear model with log2-transformed response values and adjusted by covariates including age, sex, PC1, PC2, and RIS medication history (duration, daily dose). The obtained *p* values were adjusted for multiple testing.

LD analysis was performed with PLINK v1.9 with an r^2^ threshold of 0.5 and a window of 1,000 Kb. LD figures were generated for markers with significant associations (*p*-value <0.05) using HaploView v20.0.1 (Barrett et al., 2004), where blocks were defined by solid spine (SS).

## 3 Results

### 3.1 Study population

Of the 110 samples from pediatric patients with ASD who underwent clinical and psychological evaluations, 7 individual samples were excluded from genotyping for not meeting the DQC criteria, and 8 samples were excluded due to a call rate of <90% in the genotyping results. Furthermore, 6 patients who did not provide plasma samples for drug concentration determination were excluded. The average QC call rate for the passing samples was 99.2%.

Supplementary Table S1 displays the demographics and clinical criteria of the 89 patients in our study population. Eighty-three (93.3%) patients received RIS monotherapy. The majority of patients were male (N = 67, 75.3%), with a mean age of 9 (standard deviation (SD) = 4.1) years. The median RIS dose was 0.75 (interquartile range (IQR): 0.5–1.5) mg/day, with a median treatment duration of 21.5 (IQR: 3.23–57.9) months. Thirty-two (36%) patients also received concomitant psychotropic medications, primarily psychostimulants and melatonin. The median concentration of RIS was 0.56 ng/mL (IQR: 0.3–2.4) and that of 9-OH-RIS was 7.02 ng/mL (IQR: 2.4–13.4), while the active moiety concentration was 8.18 ng/mL (IQR: 2.8–16.4). The RIS/9-OH-RIS concentration ratio was 0.14 (IQR: 0.07–0.23).

### 3.2 Selection of PharmacoFocus PGx markers

A total of 8319 probeset markers were obtained via the PharmacoFocus Array, of which 2218 markers were identified as PGx markers. In the process of filtering 2218 PGx markers with the QC parameters, 100 (4.5%) markers were removed due to a global genotyping call rate of less than 95%. Of the remaining 2118 markers, 1,378 (62.13%) markers were excluded from further analysis because their MAF was less than 5%. Furthermore, 8 (0.4%) markers on the X chromosome and 10 (0.45%) markers with HWE *p* values <0.001 were omitted.

As a result, 722 (32.6%) of 2218 PGx markers were included in the association analysis. The average call rate of the 722 selected markers was 99.7%. The PCA plot did not show any clear clusters, indicating the absence of strong subpopulation stratification (Supplementary Figure S1).

### 3.3 Association of PGx variants with RIS PK parameters in the ASD cohort

A total of 27 PGx variants in 20 genes were demonstrated by PLINK software to have a significant association (*p*-value <0.01) with various RIS PK parameters measured in plasma, including RIS, its metabolite (9-OH-RIS), total active moiety, and RIS/9-OH-RIS metabolic ratio (results are displayed in Tables 1–4, respectively). The PGx markers are arranged in the tables according to their strength of association with the response variable. The direction of impact (DOI) of each individual PGx marker was positive or negative, indicating either increasing or decreasing an RIS PK measure, and is presented in its specific table.

**TABLE 1:** Top PGx markers associated with RIS plasma levels at a minimum *p* < 0.01.

Marker name	Associated gene	Chr.	rsID	MAF	OR (95% CI)	*p*-value	DOI
FDPS_c.-1-98T>G	FDPS	1	rs2297480	0.21	1.412 (1.149–1.737)	0.00166	+ve
ADRA2A_c.*427A>G/T	ADRA2A	10	rs553668	0.152	1.468 (1.154–1.867)	0.00260	+ve
TPMT_c.141-101A>T	TPMT	6	rs12529220	0.461	1.287 (1.093–1.514)	0.00341	+ve
TPMT_c.366 + 58T>C	TPMT	6	rs2518463	0.461	1.287 (1.093–1.514)	0.00341	+ve
HLA-DPB1:c.313A>G(Met105Val)	HLA-DPB1	6	rs1042151	0.225	1.334 (1.105–1.612)	0.00386	+ve
CYP2C19_41295G>A	CYP2C19	10	rs4494250	0.253	0.737 (0.604–0.9012)	0.0046	−ve
CYP2C18_c.*31C>T(3′UTR)	CYP2C18	10	rs2860840	0.253	0.737 (0.604–0.9012)	0.0046	−ve
NAT2_c.-594G>C(5′UTR)	NAT2	8	rs4271002	0.073	1.867 (1.224–2.848)	0.0057	+ve

Abbreviations: Chr., chromosome number; DOI, direction of impact; MAF, minor allele frequency.

**TABLE 2:** Top PGx markers associated with 9-OH-RIS plasma levels at a minimum *p* < 0.01.

Marker name	Associated gene	Chr.	rsID	MAF	OR (95% CI)	*p*-value	DOI
CYP2C8*1B_-271C>A(5′UTR)	CYP2C8	10	rs7909236	0.101	0.3571 (0.1886–0.6762)	0.0023	−ve
ABCC3_c.3890G>A(R1297H)	ABCC3	17	rs11568591	0.0787	0.3196 (0.1502–0.6804)	0.0042	−ve
CYP2C8_1982A>G	CYP2C8	10	rs2275622	0.18	0.288 (0.156–0.532)	0.0052	−ve
HLA-G(rs66554220)	HLA-G	6	rs66554220	0.43	1.9811 (1.2277–3.1968)	0.0067	+ve

Abbreviations: Chr., chromosome number; DOI, direction of impact; MAF, minor allele frequency.

**TABLE 3:** Top PGx markers associated with total active moiety plasma levels at a minimum *p* < 0.01.

Marker name	Associated gene	Chr.	rsID	MAF	OR (95% CI)	*p*-value	DOI
ADRA2A_c.*427A>G/T	ADRA2A	10	rs553668	0.152	1.678 (1.233–2.283)	0.0016	+ve
CYP2E1*7B_c.-71G>T(5′UTR)	CYP2E1	10	rs6413420	0.157	0.611 (0.44–0.847)	0.0043	−ve
HLA-A(rs1061235)	HLA-A	6	rs1061235	0.121	1.532 (1.15–2.04)	0.0048	+ve
CRHR2(rs7793837)	CRHR2	7	rs7793837	0.368	1.454 (1.124–1.882)	0.0059	+ve
MTHFR_c.665C>T(Ala222Val)	MTHFR	1	rs1801133	0.129	0.602 (0.416–0.872)	0.0090	−ve

Abbreviations: Chr., chromosome number; DOI, direction of impact; MAF, minor allele frequency.

**TABLE 4:** Top PGx markers associated with the RIS/9-OH-RIS metabolic ratio at a minimum *p* < 0.01.

Marker name	Associated gene	Chr.	rsID	MAF	OR (95% CI)	*p*-value	DOI
UGT2B17_c.*317A>T(3′UTR)	UGT2B17	4	rs78998153	0.26	1.363 (1.185–1.568)	6.77 × 10^−5^	+ve
CYP2D6_-2178G>A(5′UTR)	CYP2D6	22	rs28360521	0.13	1.447 (1.215–1.724)	0.0001	+ve
CYP2D6_2098A>G	CYP2D6	22	rs2267447	0.0734	1.705 (1.314–2.213)	0.0002	+ve
CYP2D6_-1426C>T(5′UTR)	CYP2D6	22	rs28588594	0.0734	1.705 (1.314–2.213)	0.0002	+ve
CYP2D6_-1000G>A(5′UTR)	CYP2D6	22	rs1080989	0.0734	1.705 (1.314–2.213)	0.0002	+ve
CYP2D6_100C>T(P34S)	CYP2D6	22	rs1065852	0.0734	1.705 (1.314–2.213)	0.0002	+ve
CYP2D6*4_1847G>A(SpliceDefect)	CYP2D6	22	rs3892097	0.0514	1.887 (1.342–2.652)	0.0006	+ve
HLA-DRB1(rs9272346)	HLA-DRB1	6	rs9272346	0.393	0.792 (0.679–0.925)	0.0046	−ve
CDA_c.435C>T(T145 = )	CDA	1	rs1048977	0.18	1.299 (1.074–1.571)	0.0091	+ve
CYP1A1_c.-27 + 606G>T	CYP1A1	15	rs2606345	0.309	1.251 (1.063–1.472)	0.0092	+ve

Abbreviations: Chr., chromosome number; DOI, direction of impact; MAF, minor allele frequency.

Additional PGx markers that revealed potential associations with the RIS PK parameters with a minimum of *p* < 0.05 are presented in Supplementary Tables S2–S5. Nongenetic confounding variables, including age, sex, self-identified ethnicity, RIS dosage, treatment duration, and concomitant medications, were not significant in any of the models of RIS PK parameters.

**FIGURE 1:**
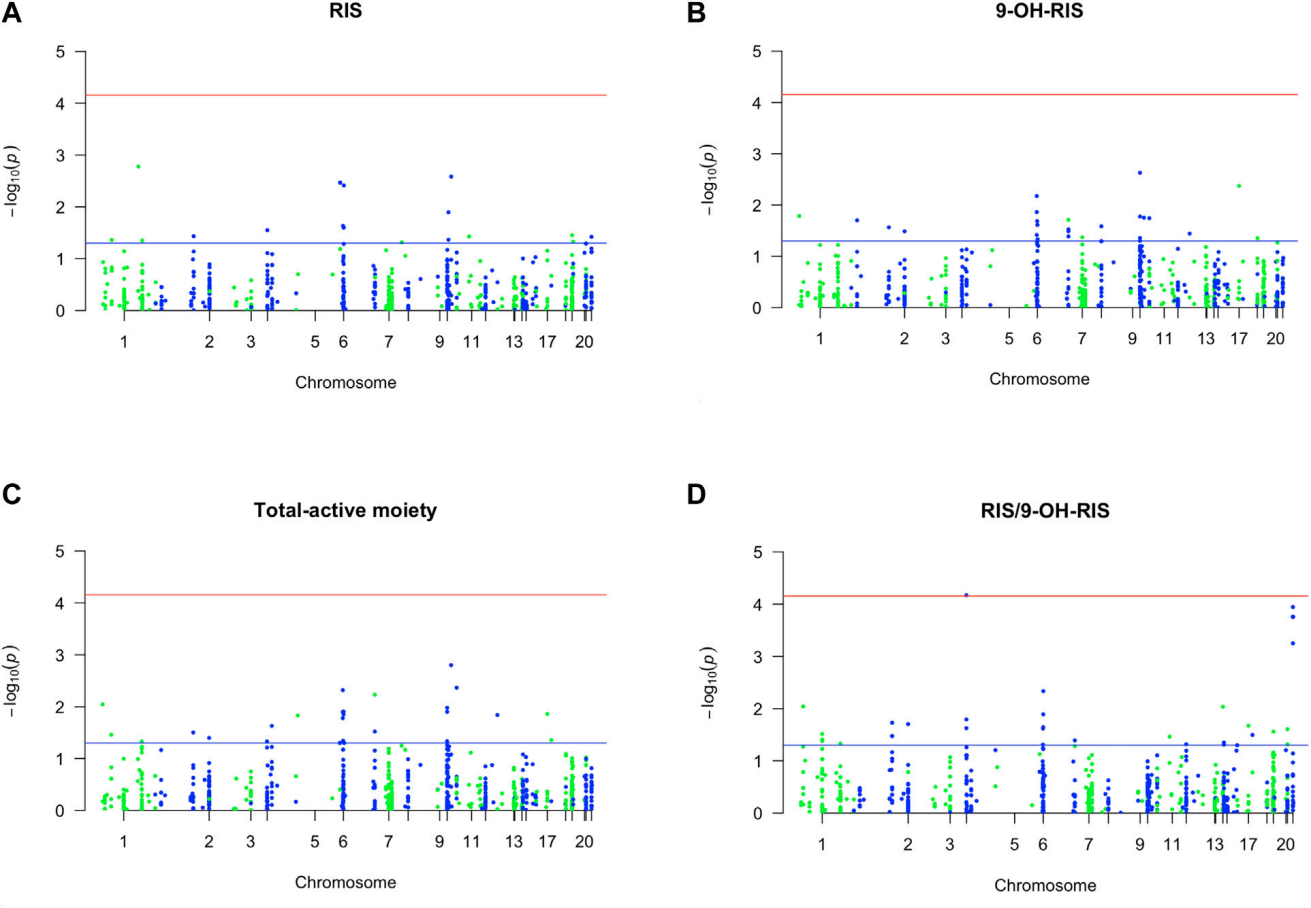
Manhattan plot of associations of RIS exposure parameters with 720 PGx PharmacoFocus markers. The horizontal x-axis represents the chromosomal position; the vertical y-axis represents–log_10_ P from the linear regression. The red horizontal line represents the significance level of *p* = 7.0 × 10^−5^ after Bonferroni correction. The horizontal blue line represents the significance level *p* = 0.05. **(A)** PGx variants of RIS exposure. **(B)** PGx variants of 9-OH-RIS. **(C)** PGx variants of the total active moiety. **(D)** PGx variants of metabolic ratio (RIS/9-OH-RIS).

First, out of all 722 included variants, only 8 markers in 7 genes were identified as top PGx markers for the RIS plasma level with *p* < 0.01 (Table 1; Figure 1A). Specifically, two SNPs (1 in *CYP2C19* (rs4494250) and 1 in *CYP2C18* (rs2860840)) were negatively correlated with the plasma level of RIS. However, the other top identified SNPs in five genes (*FDPS*, *ADRA2A*, *TPMT*, *HLA-DPB1*, and *NAT2*) were positively associated, indicating a greater effect on the RIS concentration estimates (*p* < 0.01). An additional 10 novel markers (i.e., not within the known RIS metabolic pathway) (Whirl-Carrillo et al., 2021) were identified to be associated at the level of *p* < 0.05, as shown in Supplementary Table S2. However, only one SNP (CYP2D6*4_1847G>A, splice site variant) in the *CYP2D6* gene, known to be primarily involved in the established RIS metabolic pathway (Whirl-Carrillo et al., 2021), was identified as significant at the level of *p* < 0.05 (*p* = 0.04). Although it had a low prevalence of 5% among the study sample, it was found to increase RIS levels by 1.5-fold (Supplementary Table S2).

Second, 4 PGx variants in 3 genes were found to be strongly associated with the level of the RIS metabolite (9-OH-RIS) at *p* < 0.01 (Table 2; Figure 1B). Interestingly, 2 of these SNPs are located in the *CYP2C8* gene, which encodes a novel CYP450 enzyme [CYP2C8] that was not previously known to influence the metabolism of RIS. The other 2 variants were identified in two different genes (*ABCC3* and *HLA-G*). However, at the level of *p* < 0.05, a total of 22 supplementary PGx markers in 17 genes were shown to have either a positive or negative effect on the metabolite concentration in plasma (Supplementary Table S3). Notably, among these secondary markers, 2 SNPs in the *CYP2C9* gene positively impacted the concentration of 9-OH-RIS by a 1.7-fold increase, reflecting the potential of the CYP2C9 enzyme to play an important role in the conversion of RIS to this metabolite.

Third, 5 markers in 5 different genes were shown to be significant in determining the total active moiety at the level of *p* < 0.01 (Table 3; Figure 1C). Only one PD marker (ADRA2A_c.*427A>G/T) was found to simultaneously enhance the exposure of RIS (Table 1) and the total active moiety at the level of *p* < 0.01 (Table 3). In the current study analyses, none of the metabolic PGx markers that affected RIS (Table 1) or 9-OH-RIS levels (Table 2) were simultaneously observed to have a significant impact on the total active moiety at the level of *p* < 0.01. This result indicated the possibility of their involvement in the conversion of RIS and 9-OH-RIS to other inactive metabolites.

Fourth, 10 SNPs in 5 genes were revealed to have a highly significant impact on the RIS/9-OH-RIS metabolic ratio (Table 4; Figure 1D). Notably, 6 SNPs (rs28360521, rs2267447, rs28588594, rs1080989, rs1065852, and rs3892097) in the *CYP2D6* gene (preliminarily identified as the main metabolic enzyme guiding the conversion of RIS to 9-OH-RIS) were observed to significantly influence the metabolic ratio with an average OR = 1.7 (Table 4); no considerable effects related to these markers were found on the individual estimates of RIS, 9-OH-RIS, or the total active moiety plasma concentrations. Several other supplementary PGx variants were also shown to have an influence on the metabolic ratio but at a lower threshold (*p* < 0.05), as depicted in Supplementary Table S5.

### 3.4 Linkage disequilibrium analysis

**FIGURE 2:**
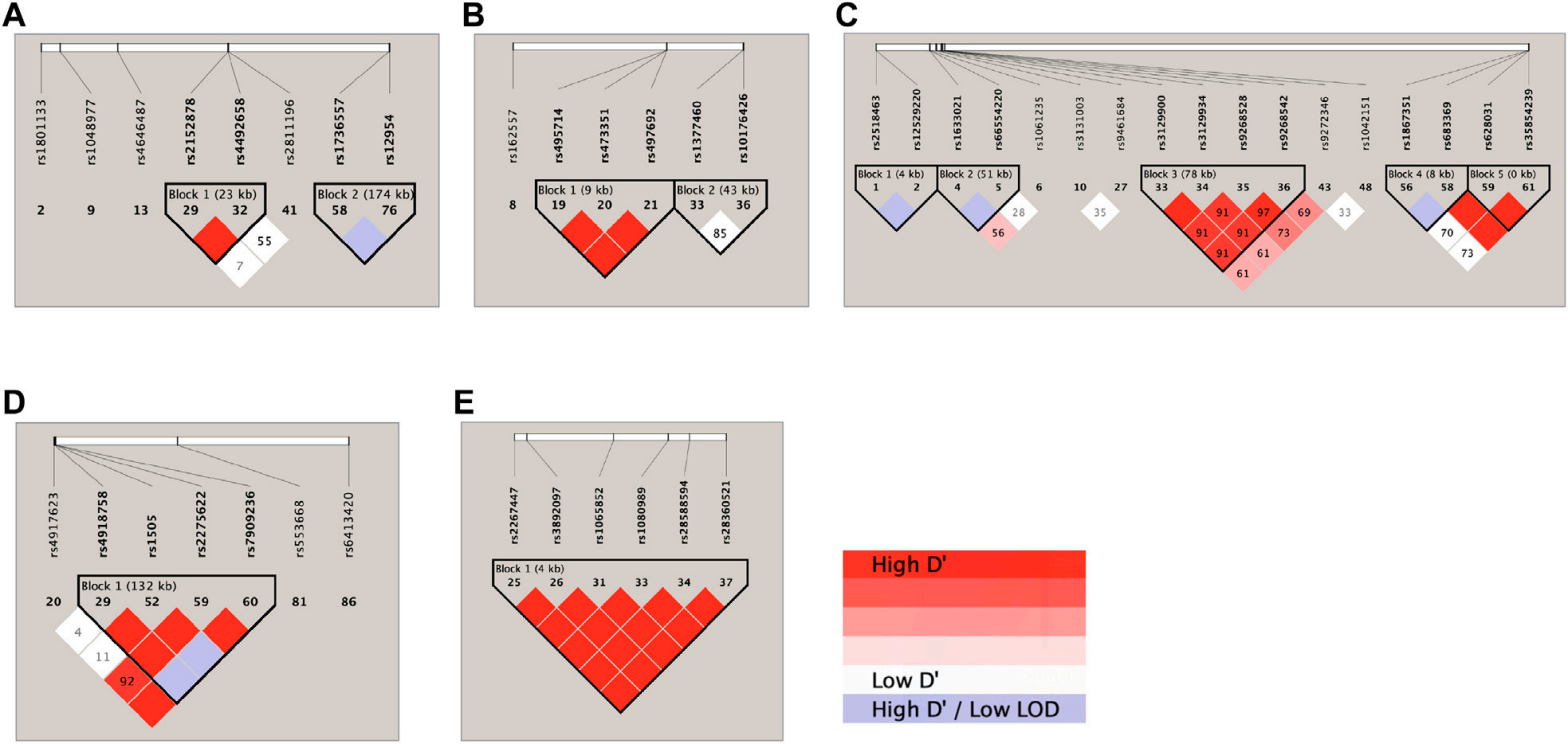
Haploview linkage disequilibrium map of SNPs associated with responses: **(A)** chromosome 1; **(B)** chromosome 2; **(C)** chromosome 6; **(D)** chromosome 10; and **(E)** chromosome 22. Pairwise linkage disequilibrium (D′) values are given in blocks for each SNP combination. Red indicates high D′ values; white indicates low D′; and violet indicates high D′ with a low logarithm of odds (LOD).

LD analysis of the genetic markers that were associated with the 4 RIS PK parameters at the level of *p* < 0.05 is shown in Figure 2. Accordingly, on chromosome 1 (Figure 2A), *DPYD* rs2152878 and rs4492658 were observed to be in strong LD, while *FMO3* rs1736557 and *FMO1* rs12954 were noted to be likely in LD. On chromosome 2 (Figure 2B), *ABCB11* rs495714, rs473351, and rs497692 were found to be in strong LD. On chromosome 6 (Figure 2C), *TPMT* rs2518463 and rs12529220 were observed to be likely in LD; *HLA-A* rs1061235 and intergenic *HCG4* (rs1633021) and *HLA-G* (rs66554220) were noted to be likely in LD; *HLA-DQA1-AS* rs3129900, rs3129934, and rs9268542 and *HLA-DQA1* rs9272346 were in strong LD; *SLC22A1* rs1867351 and rs683369 were likely in LD; and *SLC22A1* rs683369, rs628031, and rs35854239 were in strong LD. On chromosome 10 (Figure 2D), *CYP2C9* rs4918758 and rs1505 and *CYP2C8* rs2275622 and rs7909236 were in strong LD and likely in LD with *CYP2C19* rs4917623. On chromosome 22 (Figure 2E), 6 *CYP2D6* SNPs (rs2267447, rs3892097, rs1065852, rs1080989, rs28588594, and rs28360521) were found to be in strong LD.

## 4 Discussion

In this pharmacogenetic study, use of the Axiom PharmacoFocus Array platform revealed various novel PGx markers highly associated with RIS exposure in Saudi children with ASD. The key finding of this exploratory focused study is that most of the PGx markers that showed a prominent impact on various RIS PK parameters (27 out of 722 PGx variants examined) were not located within the genes involved in the classical RIS PK pathway, as previously defined by *in vivo* studies (Whirl-Carrillo et al., 2021).

### 4.1 PGx markers encoding phase I metabolic enzymes

#### 4.1.1 CYP2C8 and CYP2C9

CYP2C8 is a phase I metabolizing enzyme that has been recently described by PharmGKB as a very important pseudogene (VIP) (Whirl-Carrillo et al., 2021). Interest in CYP2C8 emerged for several reasons, such as its central role in the biotransformation of structurally dissimilar compounds and endogenous molecules, attributed to its ability to bind divergent substrates without extensive conformational changes (Lai et al., 2009); its wide expression in body tissues other than hepatocytes (Sjostedt et al., 2020; Karlsson et al., 2021); more updates in identification of its substrates and inhibitors; and advanced knowledge in characterization of its SNPs and star alleles (Gaedigk et al., 2022). Additionally, the *CYP2C8* gene is positioned on chromosome 10q24 in the *CYP2C* gene cluster (centromere-*CYP2C18*-*CYP2C19*-*CYP2C9*-*CYP2C8*-telomere), and given the proximity of *CYP2C8* and *CYP2C9*, LD was previously reported between these genes (Yasar et al., 2002). Interestingly, the present exploratory study revealed strong LD between 2 *CYP2C8* (rs7909236 and rs2275622) and 2 *CYP2C9* (rs4918758 and rs1505) SNPs, all of which were shown to have significant individual associations with 9-OH-RIS plasma levels with decreasing (*CYP2C8* pair) and increasing impacts (*CYP2C9* pair). Only the *CYP2C8* pair was associated with a decreased total active moiety, possibly indicating increased metabolism of 9-OH-RIS. This assumption may be supported by evidence from previous studies indicating that rs7909236 (-271C>A SNP designated as CYP2C8*1B) is associated with normal enzyme function compared with wild-type (Bahadur et al., 2002; Yasar et al., 2002; Rodríguez-Antona et al., 2007). While this *CYP2C8* SNP was reported to be absent in Africans, its prevalence in our population (10%) was similar to that in Asians but lower than that in Caucasians (23%) (Bahadur et al., 2002). The other *CYP2C8* SNP (rs2275622) (18% in our population) is a variant that was less commonly reported in clinical studies with contradictory functional effects (associated with higher or lower enzymatic activity) depending on the substrate (Kirchheiner et al., 2008; Grau et al., 2009).

Collectively, the observed negative impact of CYP2C8 on 9-OH-RIS and the total active moiety support the assumption that this enzyme simultaneously acts on RIS and 9-OH-RIS, with a greater reduction in the RIS level (possibly highlighting the influence of CYP2C8 on a second RIS metabolic pathway to an alternative metabolite).

However, our observed increase in 9-OH-RIS plasma levels associated with the 2 *CYP2C9* variants (both designated as a CYP2C9*1 allele with normal function) (Gaedigk et al., 2017) could be linked to increased RIS metabolism to 9-OH-RIS mediated by the CYP2C9 enzyme, presumably as a minor non-CYP2D6 pathway. In contrast, previous studies with smaller sample sizes have failed to reveal any CYP2C9 impacts on RIS PK parameters (Llerena et al., 2004; Cabaleiro et al., 2014).

Additional studies are needed to explain the relative contributions of CYP2C8 and CYP2C9 to RIS metabolism and the power of simultaneously existing *CYP2C8*/*CYP2C9* haplotypes to predict RIS efficacy and safety before implementation in clinical practice.

#### 4.1.2 CYP2C19 and CYP2C18

Two SNPs from two genes encoding different CYP450 enzymes (1 in *CYP2C19* (rs4494250) and 1 in *CYP2C18* (rs2860840)) were observed to equally contribute to reduced concentrations of RIS among carriers compared to noncarriers. Unfortunately, the functional status of those two SNPs was not defined in the known resources of CYP2C19 or CYP2C18 Allele Definition Tables (Whirl-Carrillo et al., 2021; Gaedigk et al., 2022). However, both genes are within the CYP2C subfamily, which is responsible for the metabolism of various drugs, including warfarin, escitalopram and omeprazole; however, the role of CYP2C18 in drug metabolism in general remains unclear (Goldstein and de Morais, 1994). Interestingly, the two genes are closely located on the same chromosome, and previous evidence in Japanese subjects suggested complete linkages between the mutated alleles of *CYP2C18* and *CYP2C19* (Kubota et al., 1998). Furthermore, our observation of lower RIS concentrations among autistic children carrying rs2860840 (CYP2C18_c.*31C>T(3′UTR)) reinforces a recent novel finding by Bråten et al. (2021), indicating that increased CYP2C19-dependent escitalopram metabolism leads to decreased concentrations in *CYP2C19* NMs (*1/*1), similar to levels obtained in patients classified as *CYP2C19* UMs (*17/*17) or RMs (*1/*17). The latter findings were mainly attributed to the liver enhancer effect induced by only one SNP, *CYP2C18* (rs2860840), which was simultaneously carried by the *CYP2C19* NMs. This novel SNP finding was later examined in Native American cohorts, and due to its high frequency and clinical implications for the treatment of >20 drugs with official annotations for *CYP2C19* polymorphisms, it was recommended to add this SNP to the PGx practice panel to reveal the mismatching between *CYP2C19*-predicted and exposure-substantiated *CYP2C19* metabolic phenotypes (Fernandes et al., 2023).

Our study is the first to report on the significant individual and combined impact of two SNPs in *CYP2C19* and *CYP2C18* on reducing RIS plasma levels, which could lead to infectiveness and require a higher dosage to achieve the RIS target therapeutic range. In contrast, Cabaleiro et al. (2014) reported an increased chance of RIS-induced neurologic manifestations among *CYP2C19* NM healthy individuals compared to *CYP2C19* PMs. However, in the same study, no significant impact of *CYP2C19* polymorphisms was found on RIS or 9-OH-RIS PK parameters. Whether this finding could be attributed to the absence of both SNPs identified in our study as enhancing the metabolism of RIS or the smaller sample size in the Cabaleiro et al. (2014) study remains to be examined in future large-scale studies.

#### 4.1.3 CYP2D6

In the classical RIS metabolic pathway (Whirl-Carrillo et al., 2021), CYP2D6 is the primary enzyme involved in RIS metabolism to 9-OH-RIS. The current work reinforced this fact by revealing that 6 mutated *CYP2D6* PGx variants have a significant positive impact on the RIS/9-OH-RIS metabolic ratio, which indirectly reflects a substantial increase in the plasma bioavailability of RIS in comparison to its major metabolite. Notably, all these SNPs were in complete LD in our autistic children. However, none of these variants were shown to have an influence on the individual estimates of RIS, RIS metabolite, or total active moiety, which may reflect the presence of other, undiscovered non-*CYP2D6* variants (for example, as discussed earlier, *CYP2C8/9* haplotypes) that could further modulate global exposure to and the efficacy and safety of RIS therapy in ASD. This finding is consistent with a similar observation in an exploratory study in Thai children with ASD, which revealed that three *CYP2D6* variants existing in strong LD significantly influenced the metabolic ratio but not the discrete measurements of RIS, 9-OH-RIS, or total active moiety in plasma (Medhasi et al., 2016).

#### 4.1.4 CYP2E1

CYP2E1 is a member of the CYP450 family that metabolizes relatively few prescription drugs but is better known for the metabolism of toxins and procarcinogens (Hayashi et al., 1991). Several *CYP2E1* SNPs in the promoter and intronic regions have been identified; however, luciferase promoter studies have shown that polymorphisms in the *7 haplotype, in particular the rs6413420 variant, increase *CYP2E1* transcription (Huang et al., 2012). Interestingly, the current study revealed a significant impact of this SNP (15.7% prevalence) in reducing the total active moiety (at the level of *p* < 0.01) and 9-OH-RIS (*p* = 0.018) up to 0.6- and 0.44-fold, respectively, compared to noncarriers. Consistent with this finding, two previous studies highlighted the role of *CYP2E1* SNPs in the etiology of schizophrenia and RIS treatment outcomes in the Chinese population (Huo et al., 2012; Shi et al., 2017). Of note, 5 *CYP2E1* SNPs (including *5, rs3813867 and rs2031920) were associated with increased total active moiety, suggesting lower enzyme activity (Huo et al., 2012). However, rs2515641 in *CYP2E1* was found to be significantly related to nonresponse to RIS treatment for schizophrenia in a Chinese cohort (*p* = 0.007) (Shi et al., 2017). Additionally, molecular genetic studies found that DNA methylation levels of *CYP2E1* in the placenta were associated with children later diagnosed with ASD (Zhu et al., 2019; Bahado-Singh et al., 2022). Consistent with this finding, some molecular expression studies in inflammatory-mediated gastrointestinal diseases reported increased levels of CYP2E1 (122%) (Effinger et al., 2019), which strengthens the evidence of the role of the gut–brain axis in ASD etiology (Lombardi et al., 2023; Morton et al., 2023) and its correlation to our observation of reduced total active moiety and 9-OH-RIS levels in the study.

#### 4.1.5 CYP1A1

The *CYP1A1* gene encodes the CYP1A1 enzyme, which is a member of the CYP1A subfamily and is responsible for the metabolism of diverse substrate molecules, such as sex hormones, caffeine, therapeutic drugs, environmental pollutants, toxins, and carcinogens. This diversity of CYP1A1 functions implies its involvement in numerous biological pathways (Kukal et al., 2023). Individual divergences in CYP1A1 expression and activity not only are attributed to genetic polymorphisms in *CYP1A* genes but can also be up- or downregulated through the interaction of environmental and endogenous physiological factors (Kukal et al., 2023). According to our association analysis, only one *CYP1A1* SNP (rs2606345), which was highly prevalent in our ASD population (30.9%), had a significant positive impact on the metabolic ratio. In PGx studies of other medications, this variant produced controversial findings regarding its influence on enzyme activity (Whirl-Carrillo et al., 2021). Despite the SNP’s position in the intronic region of the *CYP1A1* gene, some PK studies reported decreased function (Allegra et al., 2016; Talwar et al., 2016), while others reported a gain of function (Grover et al., 2010; Cusato et al., 2014; Allegra et al., 2017). Therefore, it is quite challenging to interpret from current data the actual contribution of this *CYP1A1* polymorphism to the elevated metabolic ratio (increased RIS or decreased 9-OH-RIS levels in plasma), particularly because it is not directly involved in the primary RIS metabolic pathway. Moreover, this enzyme function was reported to be modified by a wide range of downstream modifications (genetic or epigenetic) and environmental factors that function together to alter the expression of the underlying genetic variant, leading to the ultimate biological response (Ye et al., 2019; Xu et al., 2023). Notably, another *CYP1A1* SNP (rs1048943) was highly prevalent in Thai children with ASD (30.3%) (Sukasem et al., 2016), indicating that further molecular studies are needed to define the mechanisms connecting the *CYP1A1* polymorphism to ASD and to modulate RIS PK in this disease population.

#### 4.1.6 CDA

The *CDA* gene encodes the cytidine deaminase enzyme, which catalyzes the irreversible hydrolytic deamination of cytidine and deoxycytidine to uridine and deoxyuridine, respectively. It is one of numerous deaminases responsible for preserving the cellular pyrimidine pool. It is known that certain drugs can be rapidly metabolized by the CDA enzyme, which can affect their bioavailability and efficacy (Lavelle et al., 2012). In the context of chemotherapy, CDA plays a significant role (Abbaspour et al., 2023), since it metabolizes several chemotherapeutic drugs, including gemcitabine (Pellicer et al., 2017). Gemcitabine interferes with DNA synthesis and replication either by inhibiting enzymes involved in the synthesis of nucleic acid precursors or by misincorporation of nucleic acids into DNA or RNA macromolecules (Ciccolini et al., 2016). Studies have shown that the activity of the CDA enzyme can be a predictive biomarker in gemcitabine-treated cancer patients. Patients with lower CDA activity had significantly longer survival compared to patients with higher CDA activity (Soo et al., 2009). The current data suggested that one *CDA* variant (CDA_c.435C>T(T145 = ), rs1048977) was a strong predictor for a higher RIS/9-OH-RIS metabolic ratio. However, to date, there is no specific information in the medical literature about the interaction between CDA and RIS (Whirl-Carrillo et al., 2021). In the context of psychiatry and ASD, there is ongoing research into the role of various enzymes and their potential impact on these conditions. Some studies have demonstrated that *CDA* gene expression in the brain is associated with certain psychiatric disorders by creating DNA mutations via deamination of cytosine bases, which results in uracil (Gavin et al., 2012; Guidotti and Grayson, 2014). The same studies highlighted antipsychotics, including RIS, as potential targets in altering DNA methylation profiles in the brain. One study found that salivary immunoglobulin A (IgA) levels were significantly decreased in patients with ASD, and this correlated with bacteria-induced downregulation of the polymeric immunoglobulin receptor (Pigr) in salivary glands (Gong et al., 2021). However, this study did not specifically mention the CDA enzyme. Another study discussed the role of activation-induced CDA (AID) in the adaptive immune system and its potential implications in various diseases (Rios et al., 2020). However, it did not specifically link AID to autism or psychiatric disorders. While these studies provide valuable insights, more research is needed to fully understand the complex interactions among CDA, AID, and ASD etiology. Subsequently, it is important to investigate how genetic polymorphisms affecting *CDA* could act as markers for RIS clinical outcome (i.e., toxicity, efficacy) in real clinical practice.

### 4.2 PGx markers encoding phase II metabolic enzymes

#### 4.2.1 FDPS

Another novel variant that was found to be associated with increased RIS levels was rs2297480 (by 1.4-fold, *p* = 0.0012) in the *FDPS* gene, which encodes farnesyl diphosphate synthase (FDPS), an essential enzyme in the mevalonate pathway (cholesterol biosynthesis) (Göbel et al., 2020). The *FDPS* gene is implicated in various diseases, including neuropsychiatric disorders such as ASDs (Segatto et al., 2019; Lin et al., 2023). Additionally, as an RNA-binding protein, FDPS is also involved in transcriptional and post-transcriptional regulation of many enzymes (Wang L. et al., 2023). A recent *in silico* analysis revealed that *FDPS* is an overlapping gene that is involved in CNS disorders and is simultaneously associated with the encoding of enzymes in the lipid and cholesterol metabolic pathways (Ang and Moon, 2022). Of note, antipsychotic drugs were observed to result in upregulated expression of the genes involved in cholesterol biosynthesis (Le Hellard et al., 2008), which was suggested as a potential causal pathway for their role in the pathogenesis of neuropsychiatric disorders (Zhou et al., 2021) as well as their subsequent induced adverse metabolic effects (Le Hellard et al., 2008). However, according to the present association study, how *FDPS* polymorphisms and expression modulate RIS exposure and response in ASD patients and *vice versa* remain unknown. However, our results highlight the need for further investigation of the pathways underlying this gene-disease-drug interaction.

#### 4.2.2 TPMT

The *TPMT* gene encodes the thiopurine S-methyltransferase enzyme, which plays a crucial role in the metabolism of thiopurine drugs (Lee et al., 1995). Moreover, it is dependent on the S-adenosylmethionine (SAM) methyl donor substrate in the methionine pathway (Weinshilboum, 2006). The TPMT enzyme is involved with other conjugation enzymes in phase II detoxification, where liver cells add a substance (such as cysteine, glycine, methyl or a sulfur molecule) to a toxic chemical or drug to make it less harmful and easier for the body to excrete (Zhang, 2011). It has also been implicated in the metabolism of other aromatic and heterocyclic sulfhydryl compounds (Woodson and Weinshilboum, 1983).

Several pharmacogenetic studies have demonstrated that certain polymorphism-induced mutations in the *TPMT* gene result in completely undetectable TPMT enzyme activity, leading to life-threatening adverse events associated with even normal doses of anticancer drugs, such as azathioprine, cyclosporine, and daunorubicin (Wang et al., 2010). However, TPMT is not involved in the direct metabolic pathway (Phase I) of RIS (Whirl-Carrillo et al., 2021). In our study, two *TPMT* intronic SNPs (rs12529220 and rs2518463; both known to express the normal functional *TPMT* allele *1), with a prevalence of 46.1% among Saudi autistic children, were likely to be in LD at chromosome 6. Both were associated with a substantial increase in RIS levels (1.3-fold). Consistent with this finding, a previous RIS PK study in normal volunteers demonstrated significantly higher 9-OH-RIS plasma levels in *1/*1 genotype participants in comparison to mutant genotype carriers (*1/*2,*1/*3C,*1/*3A) (Cabaleiro et al., 2014). Another earlier study reported an association of decreased TPMT activity (mutant genotypes) with olanzapine-induced fatigue and dizziness in healthy volunteers with no significant impact on any of its PK parameters (Cabaleiro et al., 2013). However, no other data are available on their association with the PK of RIS or other antipsychotics (Whirl-Carrillo et al., 2021); therefore, it is challenging to provide a satisfactory interpretation. Further studies are needed to explore the impact of *TPMT* polymorphisms on chronic RIS therapy in ASD.

#### 4.2.3 NAT2

The *NAT2* gene encodes N-acetyltransferase 2 (arylamine N-acetyltransferase), which is a typical xenobiotic metabolizing enzyme (Ackenheil and Weber, 2004) responsible for acetylation as a phase II conjugation reaction. In previous studies, NAT2 was identified to play a role in the metabolism of benzodiazepines (Camargo et al., 2023) and also hypothetically plays a role in the metabolism of some antipsychotics (Ackenheil and Weber, 2004). To date, 88 SNPs have been identified within the *NAT2* gene that can affect NAT2 function by resulting in reduced enzyme stability or altered affinity for a substrate. *NAT2* genotypes can be divided into three subgroups: “slow acetylator” (two slow alleles), “intermediate acetylator” (1 slow and 1 rapid allele), and “rapid acetylator” (2 rapid alleles, occasionally referred to as “fast”). Out of 38 *NAT2* SNPs examined in our exploratory study via the PharmacoFocus array, NAT2_c.-594G>C (5′UTR) (rs4271002) had a significant impact, increasing the RIS plasma level by 1.867-fold compared to noncarriers. Therefore, it is expected that carriers of this mutant allele may require dose modification to avoid RIS-induced adverse effects. Importantly, this is a novel *NAT2* variant that is not a part of any named alleles and has been shown in one study to be significantly associated with the risk of aspirin-intolerant asthma (Kim et al., 2010). According to PharmGKB, the functional consequence of this SNP is currently unknown, but it may affect transcription, and its exact role in the RIS metabolic pathway remains unclear (McDonagh et al., 2014). However, our result is consistent with the impact of *NAT2* polymorphisms in the study by Cabaleiro et al. (2014) reporting an increased incidence of RIS-induced headache among *NAT2* IM and PM healthy individuals in comparison to *NAT2* NMs.

#### 4.2.4 UGT2B17

The *UGT2B17* gene encodes uridine diphosphate glycosyltransferase 2 family, member B17, which is part of the family of UDP-glucuronosyltransferases (UGTs) (Beaulieu et al., 1996). As part of the phase II liver detoxification system, these genes are responsible for maintaining steady-state levels of a variety of substrates, including steroid hormones, by catalyzing the transfer of glucuronic acid moieties to these molecules and rendering them hydrophilic (Burchell et al., 1995). UGT2B17 is expressed not only in the small intestine and liver but also in steroid target tissues such as the breast, uterus, and prostate, where the extent of glucuronidation can be substantial (Karlsson et al., 2021), indicating its potential role in hormonally induced diseases (Wilson et al., 2004). However, molecular studies reported that UGT2B17 isoforms had a 4.4-fold higher abundance in the intestine than in the liver (Zhang et al., 2018). This fact suggested the potential of UGT2B17 to have a greater first-pass effect on its substrates when orally administered than subsequent liver metabolism, particularly in high UGT2B17-expressing individuals (Zhang et al., 2018). Additionally, in proteome studies, the UGT2B17 isoform, unlike other UGTs, was expressed in different brain regions, particularly in the cerebellum (Karlsson et al., 2021). According to the current study data, *UGT2B17* rs78998153, which had a prevalence of 26% among our ASD children, exhibited a very significant effect on RIS exposure. Indeed, after Bonferroni correction for multiple testing, this novel variant is the only PGx marker that still demonstrated a positive impact on the RIS/9-OH-RIS metabolic ratio, indicating a substantial increase in the RIS plasma circulating levels in comparison to its major metabolite. Consistently, an exploratory-based study of RIS PK in Thai children with ASD demonstrated that *UGT2B4* c.∗448A>G (rs1131878), an isoform that is more highly expressed in liver than brain tissues (Karlsson et al., 2021), was highly associated with the metabolic ratio (Medhasi et al., 2016). Additional evidence for the impact of UGTs on RIS PK can be drawn from a study in Thai children with ASD, where three SNPs indicating *UGT1A1* mutation (an isoform that is highly expressed in brain tissues (Sjostedt et al., 2020)) have shown a significant association with the RIS-induced prolactin response (Hongkaew et al., 2018). Collectively, these results highlight the need for further molecular studies to explore the correlation between various *UGT* genotypes and their induced modifications in UGT enzyme expression in the brain tissues of patients with ASD. This research topic is anticipated to enable a better understanding of altered RIS disposition in the brain and its precise dose individualization requirements under this central nervous system condition (Sheng et al., 2021).

#### 4.2.5 MTHFR

The *MTHFR* gene encodes the enzyme methylenetetrahydrofolate reductase (MTHER). MTHER is involved in a chemical reaction involving forms of the vitamin folate. Specifically, this enzyme converts 5,10-methylenetetrahydrofolate to 5-methyltetrahydrofolate. This reaction is part of the multistep process that converts the amino acid homocysteine to another amino acid, methionine. The body uses methionine to make proteins and other important compounds, such as neurotransmitters (dopamine and serotonin) (Jalgaonkar et al., 2022; Majhi et al., 2023). Individuals homozygous for the SNP rs1801133 (MTHFR_c.665C>T(Ala222Val)) have lower *MTHFR* activity than CC or CT (heterozygous) individuals and therefore are predisposed to hyperhomocysteinemia associated with lower plasma folate levels (Majhi et al., 2023). A meta-analysis conducted on the association between *MTHFR* SNPs and ASD susceptibilities indicated that *MTHFR* rs1801133 was associated with ASD in the five genetic models (Li et al., 2020).

Consistent with this, a growing body of evidence suggests that the severity of autistic symptoms as a whole may be associated with increased levels of homocysteine associated with aggravation of dopamine deficiency (Carpita et al., 2023; Dangmann, 2023; Majhi et al., 2023). According to our data, rs1801133 of *MTHFR*, despite being less prevalent in our sample (12.9%) than in another cohort of Saudi children with ASD (36%) (Arab and Elhawary, 2019), was associated with a significant decrease in 9-OH-RIS and total active moiety levels, yet no evident impact was observed on RIS plasma exposure. The decreased concentration of the active moiety and a more pronounced effect on 9-OH-RIS could be explained by several factors. 9-OH-RIS undergoes minor hepatic metabolism and is primarily excreted unchanged by the kidney (79.6%). One of the known metabolic pathways for 9-OH-RIS is mediated by oxidative *N-*dealkylation, forming the acid metabolite M1 (Citrome, 2007; Vermeir et al., 2008). Emerging evidence indicates that high levels of homocysteine may enhance several metabolic pathways, such as oxidation (oxidative stress), nitrosylation, acylation, and hypomethylation (Perna et al., 2003a; 2003b). According to drug metabolism theories, these mechanisms (except hypomethylation) are believed to produce more polar metabolites that cannot diffuse across membranes and may, therefore, be actively transported (Li et al., 2019). Therefore, enhanced 9-OH-RIS excretion linked to hyperhomocysteinemia (induced by the MTHER mutation) via oxidation is assumed to be a superior postulated mechanism. In addition, homocysteine is a sulfur-containing amino acid (Lentz, 2005), which could serve as a co-factor in the conjugation of a drug metabolite by sulphation (Chen and Tang, 2022), leading to increased facilitated excretion in urine (Pan et al., 2020). However, these hypotheses remain uncertain. Clearly, more studies are necessary to elucidate the role of homocysteine in enhancing 9-OH-RIS excretion and its clinical consequences in ASD patients.

### 4.3 PGx markers encoding transporters

#### 4.3.1 ABCC3

The *ABCC3* gene encodes a protein that is a member of the superfamily of ATP-binding cassette (ABC) transporters. These ABC proteins transport various molecules across extra- and intracellular membranes. The specific function of this transporter has not yet been determined; however, it was reported to mediate biliary and intestinal excretion of organic anions (Banach et al., 2022). The functional activity of some *ABCC3* variants has been fully examined (Singh et al., 2020). The current study revealed for the first time that a specific variant of the ABCC3 gene (ABCC3_c.3890G>A(R1297H)) had a prominent negative impact on 9-OH-RIS plasma levels, indicating decreased excretion (efflux) from target cells (hepatocytes or brain) to the bile or peripheral blood. The negative impact of this variant on RIS clinical efficacy and safety in ASD carriers warrants further investigation.

#### 4.3.2 ABCB11

The *ABCB11* gene encodes the ATP-binding cassette subfamily B member 11 protein, which is another member of the superfamily of ABC transporters. This membrane-associated protein is also named bile salt export pump (BSEP) or sister of P-glycoprotein (sPgp) (Strautnieks et al., 1998). Consistent with a previous exploratory study in Thai children with ASD (Medhasi et al., 2016), the current work detected the same 4 *ABCB11* variants (*ABCB11* rs495714, rs496550, rs473351 and rs497692), which displayed a significant reducing effect on the RIS/9-OH-RIS metabolic ratio, although at a lower rank of importance (Supplementary Table S4). Notably, our results in this population (Figure 2B) are compatible with the previous LD finding (Medhasi et al., 2016) that 3 *ABCB11* SNPs were strongly linked. This observation hypothetically indicates a more predominant influence of these mutations on reducing RIS efflux than 9-OH-RIS efflux, probably from various tissues’ cells into bile or blood. In contrast, previous candidate gene studies in Caucasian children with ASD (Correia et al., 2009; Rafaniello et al., 2017) and adults with schizophrenia (Xing et al., 2006; Kuzman et al., 2008) have linked reduced RIS efflux with other ABC transporter subtype mutations (ABCG2_c.421C>A in *ABCG2*; c.3435C>T, c.1199G>A, c.1236C>T and c.2677G>T in *ABCB1*). These inconsistent findings emphasized the importance of genome-wide exploratory studies to reveal disease-specific PGx markers of certain drugs, with priority according to clinical significance and their ethnogeographic frequencies.

### 4.4 PGx markers encoding PD receptors

#### 4.4.1 ADRA2A

One of the receptors that RIS blocks is the alpha 2A adrenoceptor (α2A-AR), which is an adrenergic receptor that responds to adrenaline and noradrenaline (Shahid et al., 2009). α2A-AR is encoded by the *ADRA2A* gene and is mainly found in the brain, where it regulates various functions, such as mood, cognition, attention, and sleep (Nyrönen et al., 2001). The role of α2A-AR in the therapeutic effects and side effects of RIS is not fully understood, but some studies have suggested that blocking this receptor may have both positive and negative consequences (Marcus et al., 2009). Additionally, other factors, such as genetic variations, drug interactions, and individual differences, may influence the response to RIS and the α2A-AR antagonism effect (Uys et al., 2017). The current association analysis revealed that *ADRA2A* SNP rs553668 carriers exhibit significantly increased RIS and total active moiety plasma levels (by 1.468- and 1.678-fold, respectively) in comparison to noncarriers. This finding could be interpreted in light of a previous study involving pheochromocytoma patients reporting that *ADRA2A* SNPs rs553668/rs521674 were associated with higher dosage requirements of α-adrenergic receptor blockers to control blood pressure (Berends et al., 2022).

Both our results and previous findings suggested that a certain degree of mutation in the α2A-AR receptor (decreased expression and/or density) mediated by these *ADRA2A* SNPs could lead to decreased drug-receptor occupancy and affinity, which could explain the subsequent higher PK exposure of any drugs targeted to antagonize it, such as that shown in our ASD patients exhibiting higher RIS plasma levels adjusted by the RIS dose. However, this assumption regarding the correlation between receptor affinity and drug plasma level remains speculative, and the functional consequences of the *ADRA2A* SNP on its receptor need to be examined. To our knowledge, to date, no other antipsychotic PGx studies have addressed the potential clinical consequences of polymorphisms of any of the genes encoding adrenergic receptors (including the *ADRA2A* gene) on efficacy and safety (Bousman et al., 2023). Therefore, it is important to investigate the current observation of significantly higher RIS plasma levels associated with *ADRA2A* polymorphisms in terms of clinical impacts in a larger cohort.

#### 4.4.2 CRHR2

Corticotropin-releasing hormone receptor 2 (CRHR2) is a protein that is encoded by the *CRHR2* gene, which is highly expressed in the choroid plexus (part of the blood–brain barrier) of the human brain and to a lesser extent in the plasma membranes of hormone-sensitive cells, including those in the gastrointestinal tract and kidney (Pal et al., 2010). CRH is a hormone secreted from the hypothalamus in response to stress, which needs to efficiently bind with the CRHR2 receptor to stimulate its effects (Grammatopoulos et al., 1999). CRF is a key hormone that is involved in the control of various body systems via its mediatory stimulation of the hypothalamic–pituitary–adrenal (HPA) axis. On the other hand, hypothalamic CRF, via its action on the HPA axis, may be partially involved in the reinforcing effects of metabolic enzymes in phases I and II (Mormede et al., 2011; Wójcikowski and Daniel, 2011; Bromek and Daniel, 2021). In addition, recently, increased activation of the HPA axis was suggested to play an important role in ASD-like social behaviors (Jacobson, 2014; Rusch et al., 2023). As CRHR2 is one of the receptors for the hormones involved in the HPA axis, decreased CRHR2 expression levels in the hypothalamus were recently suggested to increase the risk of ASD (Wang X. et al., 2023). Interestingly, the current data revealed that an intronic *CRHR2* variant (rs7793837), which mostly causes mutations in the CRHR2 protein, was significantly associated with a positive impact on the total active moiety level in the plasma of children with ASD. These findings suggest that CRH could play a complex role in drug metabolism and possibly in the clinical response to RIS therapy, and further research could clarify its significance as a PGx marker within the context of ASD. A possible explanation for this increased level of RIS and 9-OH-RIS could be attributed to decreased binding of CRH to the mutated *CRHR2* variant 3 (rs7793837), leading to its decreased functional impact on various downstream signaling pathways mediating the metabolism and excretion of both molecules. However, this hypothesis needs to be proven in further studies to elucidate its clinical impact on RIS therapy outcomes.

### 4.5 PGx markers encoding immunity proteins

Growing evidence in recent decades has highlighted the role of alterations in immune function, including heightened inflammation, anti-brain protein antibodies, and changes in T-cell and natural killer (NK) cell function in individuals diagnosed with ASD (Hsiao, 2013; Morton et al., 2023). Given that ASD may be induced by immunological or inflammatory pathological processes within the brain, GWASs have identified various associated human leukocyte antigen (HLA) risk alleles, including those related to *HLA-DPB1*, that obtained the most significant probabilities (Nudel et al., 2019; Morton et al., 2023). The current study revealed significant associations between *HLA-DPB1* (rs1042151), *HLA-G* (rs66554220), and *HLA-A* (rs1061235) and increased plasma levels of RIS, 9-OH-RIS, and total active moiety, respectively. A possible explanation for these influences could be attributed to the recently reported potential interplay between gut inflammatory processes mediated by these HLA markers and ASD incidence in children (the gut–brain axis) (Lombardi et al., 2023; Morton et al., 2023), thus leading to the postulation of pathophysiological changes in gastrointestinal permeability with subsequent alterations in RIS absorption and other possible inflammatory process-related consequences on the downregulation of the hepatic expression of its metabolizing enzymes or transporters (Effinger et al., 2019).

The latter hypothesis could be of interest for further examination in molecular expression studies involving ASD patients to confirm the impact of HLA-mediated inflammatory status on any medication’s global PK, particularly those that are indicated for chronic use, such as RIS.

### 4.6 Strengths and limitations

Our study had several strengths and limitations. The first major strength of the current study compared with earlier candidate gene studies is the employment of an exploratory focused pharmacogenetic approach with a comprehensive array platform, which enables genotyping of most known PGx markers in genes related to the exposure and clinical consequences of most drugs, as curated by PharmGKB. Second, this is the first study to describe RIS PK in a cohort of Arabic children. To date, only a few RIS PK studies have been conducted, and these have focused mostly on children of European backgrounds (Maruf et al., 2021). Third, in addition to characterization of the exposure of RIS in Arabic children based on classical pathways, the modern methodology enabled identification of genetic variants of known association with ASD etiology that could specifically modulate RIS exposure. Fourth, our hypothesis-generating approach in this study revealed several novel PD SNPs that have a significant influence on RIS exposure in ASD children rather than PK SNPs alone, which indicated the need for further population PK modeling studies in this specific population (with more extensive blood sampling at several time points) to re-estimate the other RIS PK parameters such as steady-state volume of distribution and absorption and elimination rates. Modeling studies incorporating the PD, PK, and disease variants as predictors of interindividual variabilities in RIS plasma concentrations are speculated to enable more precise dosing and individualized therapy for children with ASD. Fifth, we employed LD analysis to reveal significant haplotype approximate loci that are not obtained by single genetic variation genotyping alone (Eberle et al., 2006).

However, there are also limitations that should be acknowledged. As detailed earlier, the study revealed significant associations for several PGx variants describing novel pathways (for example, immunity markers), but most of these associations did not remain significant after correction for multiple testing. This could be due to a lack of statistical power, in addition to the large number of markers tested in this study. However, our strict limitation to *p* values of less than 0.01 (together with 95% CI interval) in the top tier findings of this cohort has added confidence in adjusting for type I error to avoid false-positive findings. In addition, concerns were raised regarding the misuse and overly conservative practice of correction for multiple testing in exploratory studies due its potential to produce false-negative conclusions for significant markers that are certainly important (type II error) (Johnson et al., 2010). Alternatively, to ideally ensure richness in datasets’ information to answer the exploratory research question (finding of innovative unanticipated associations), correction for multiple testing is statistically ill-advised, particularly if the modeling was adjusted by other justifiable techniques (such as PCs and nongenetic variables) (Rothman, 1990; García-Pérez, 2023). Second, since our study population has rarely been explored, our novel findings should be considered hypothesis generating and require validation in diverse ancestry cohorts. Third, in this study, RIS clinical outcomes reflecting effectiveness and safety were not examined; therefore, their relation to the novel variants identified in this study require further validation in future studies to confirm their utility in clinical practice with chronically treated patients with ASD. Finally, markers that failed the DQC parameters were removed from the present analysis. Some of these SNPs are related to the novel genes discovered in this study. Therefore, further association studies including these SNPs could strengthen the evidence of their related genes’ impact on RIS PK.

## 5 Conclusion

In conclusion, the study provided strong evidence of an interplay of PK (metabolic enzymes), transporters, PD (receptors), and other novel groups of genetic variants (immune markers) in determining the RIS exposure level in ASD patients. The study also demonstrates the importance of an exploratory approach via the Axiom array technique, which has contributed to more precisely revealing and simulating the complex system of the pathophysiology of RIS disposition in children with ASD, in comparison to earlier candidate gene approach studies, where relevant genes were probably not fully addressed. Additionally, there could be physiologically relevant signaling pathways for some of our novel PGx markers that have not yet been revealed, and polymorphisms in genes influencing the signal transduction of these variants could also be of interest to reveal the complicated mechanisms underlying autistic phenotypes. Therefore, future studies in a larger cohort of diverse ancestry groups could confirm our current findings and improve the knowledge base on how these PGx variants could modify the efficacy of RIS or other antipsychotics and the risk of developing side effects in a broader range of ASD phenotypes, characterized by diverse neurological alterations, which could facilitate personalized therapeutic decision-making in this complex neurodevelopmental condition. In addition, the present findings could open a state-of-the-art track for mechanistic research into genetic informers of variability in antipsychotic exposure-mediated responses, which may indicate a novel approach for drug development.

## Data Availability

The datasets presented in this study can be found in online repositories. The names of the repository/repositories and accession number(s) can be found in the article/Supplementary Material.

## Abbreviations

ADME, absorption, distribution, metabolism, and excretion; ASD, autism spectrum disorder; DOI, direction of impact; GWAS, genome-wide association study; HWE, Hardy–Weinberg equilibrium; IQR, interquartile range; LD, linkage disequilibrium; PD, pharmacodynamics; PGx, pharmacogenetic; PharmGKB, Pharmacogenomics Knowledge Base; PK, pharmacokinetics; QC, quality control; MAF, minor allele frequency; UM, ultrarapid metabolizer; NM, extensive or normal metabolizer; IM, intermediate metabolizer; PM, poor metabolizer; RIS, risperidone; 9-OH-RIS, 9-hydroxyrisperidone; SNP, single-nucleotide polymorphism.
