# Supplementary material for: Exploratory focused pharmacogenetic testing reveals novel markers associated with risperidone pharmacokinetics in Saudi children with autism
Source: Front Pharmacol. 2024 Feb 5;15:1356763. doi: 10.3389/fphar.2024.1356763 (PMC10875102; doi:10.3389/fphar.2024.1356763)
Supplement: Supplementary file 1 [file Table1.pdf]

Supplementary Table 1. Sociodemographic and clinical characteristics of the study population of 89 pediatric patients treated with RIS for ASD.

**Criteria**	**Value***
Age (years)	9.0 (4.1)
BMI (kg/m^2)	21.81 (7.6)
**Sex**	
Male	67 (75.3)
Female	22 (24.7)
**Ethnicity**	
White	78 (87.6)
Black	11 (12.4)
**Comorbid psychiatric disorders**	
Disruptive behavior	3 (3.4)
Anxiety	4 (4.5)
Eating disorders	1 (1.1)
Persistent depressive disorder (dysthymia)	1 (1.1)
ADHD	8 (9)
Daily RIS dosage (mg/day); median (range; IQR)	0.75 (5.75; 0.5-1.5)
RIS treatment duration (months); median (range; IQR)	21.5 (178.9; 3.23-57.9)
**Plasma drug levels, median (range; IQR), ng/ml**	
RIS	0.56 (10.82; 0.3-2.4)
9-OH-RIS	7.02 (46.38; 2.4-13.4)
Active moiety	8.18 (49.97; 2.8-16.4)
RIS/9-OH-RIS ratio	0.14 (1.72; 0.07-0.23)
**Plasma concentration-to-dose (C/D) ratios, median (range; IQR), ng/ml/mg**	
C/D of RIS	1 (11.98; 0.46-2.2)
C/D of 9-OH-RIS	9.25 (165.7; 2.8-15.8)
C/D of the active moiety	10.98 (170; 4.1-18.8)
**Medication history, n (%)**	
RIS monotherapy	83 (93.3)
Psychotropic medications	32 (36%)

Other antipsychotics	6 (6.7%)
Mood stabilizers	2 (2.2%)
Alpha 2 adrenergic agonists	1 (1.1)
Psychostimulants	6 (6.7%)
Antidepressants	1 (1.1)
Other (atomoxetine, buspirone, diphenhydramine, melatonin)	23 (25.8)

*Values are presented as the mean (SD), median (range; IQR), or percentage (n) %. Abbreviations: RIS, risperidone; CPZE, chlorpromazine equivalent dose.

Supplementary Table 2. Top PGx markers associated with RIS plasma levels at a minimum P < 0.05

Marker name	Associated gene	Chr.	rsID	MAF	OR (95% CI)	P value	DOI
ABCC2_c.-24C>T(5'UTR)	ABCC2	10	rs717620	0.107	1.444 (1.09–1.91)	0.01274	+ve
NR_002139(rs1633021)	HCG4	6	rs1633021	0.242	1.263 (1.04–1.539)	0.02330	+ve
NR_136246(rs3129900)	NR_136246	6	rs3129900	0.174	1.287 (1.04–1.597)	0.02515	+ve
NR_136245(rs3129934)	NR_136245	6	rs3129934	0.174	1.287 (1.04–1.597)	0.02515	+ve
UGT2B17_c.*317A>T(3'UTR)	UGT2B17	4	rs78998153	0.26	1.219 (1.03–1.45)	0.02819	+ve
CYP2B6_25505C>T(R487C)	CYP2B6	19	rs3211371	0.0562	0.640 (0.425–0.96)	0.03537	−ve
ABCB11_c.-8583G>A(5'UTR)	ABCB11	2	rs3770603	0.18	0.808 (0.66–0.983)	0.03684	−ve
GSTP1*B_c.313A>G(I105V)	GSTP1	11	rs1695	0.343	0.824 (0.689–0.99)	0.03733	−ve
CYP2D6*4_1847G>A(SpliceDefect)	CYP2D6	22	rs3892097	0.0514	1.537 (1.03–2.289)	0.03792	+ve
ABCC2_c.1249G>A(V417I)	ABCC2	10	rs2273697	0.303	0.818 (0.676–0.99)	0.04309	−ve
CYP4B1_c.517C>T(R173 W)	CYP4B1	1	rs4646487	0.0674	0.715 (0.519–0.98)	0.04355	−ve

Abbreviations: Chr.: chromosome number; DOI: direction of impact; MAF: minor allele frequency.

Supplementary Table 3. Top PGx markers associated with 9-OH-RIS plasma levels at a minimum P < 0.05

Marker name	Associated gene	Chr.	rsID	MAF	OR (95% CI)	P value	DOI
HLA-A(rs1061235)	HLA-A	6	rs1061235	0.121	2.099 (1.182–3.728)	0.0137	+ve
MTHFR_c.665C>T(Ala222Val)	MTHFR	1	rs1801133	0.129	0.391 (0.185–0.826)	0.0164	−ve
ADRA2A_c.*427A>G/T	ADRA2A	10	rs553668	0.152	2.209 (1.167–4.183)	0.0176	+ve
CYP2E1*7B_c.-71G>T(5'UTR)	CYP2E1	10	rs6413420	0.157	0.437 (0.224–0.854)	0.0180	−ve
CRHR2(rs7793837)	CRHR2	7	rs7793837	0.368	1.904 (1.125–3.225)	0.0194	+ve
CYP1B1_c.-2531A>G(5'UTR)	CYP1B1	2	rs162557	0.114	0.427 (0.213–0.859)	0.0199	−ve
HLA-C(rs9461684)	HLA-C	6	rs9461684	0.124	2.589 (1.180–5.682)	0.0205	+ve
NR_136245(rs3129934)	NR_136245	6	rs3129934	0.174	1.928 (1.103–3.370)	0.0244	+ve
NR_136246(rs3129900)	NR_136246	6	rs3129900	0.174	1.928 (1.103–3.370)	0.0244	+ve
NAT1*11_c.-40A>T(5'UTR)	NAT1	8	rs4986989	0.112	0.458 (0.234–0.897)	0.0260	−ve
ABCB11_c.*236G>A(3'UTR)	ABCB11	2	rs473351	0.337	1.810 (1.082–3.030)	0.0271	+ve
SLC22A1_c.1276+9_1276+16del	SLC22A1	6	rs35854239	0.292	0.604 (0.387–0.942)	0.0296	−ve
UGT1A9_c.-666C>T(5'UTR)	UGT1A9	2	rs10176426	0.135	2.063 (1.077–3.954)	0.0325	+ve
SLC22A1_c.480C>G(F160L)	SLC22A1	6	rs683369	0.0899	0.387 (0.165–0.908)	0.0325	−ve
YEATS4(rs7297610)	YEATS4	12	rs7297610	0.102	0.455 (0.221–0.936)	0.0360	−ve
HCG4(rs1633021)	HCG4	6	rs1633021	0.242	1.741 (1.041–2.911)	0.0383	+ve
SLC22A1_c.1222G>A(V408M)	SLC22A1	6	rs628031	0.298	0.621 (0.397–0.972)	0.0408	−ve
ABCB1_c.287-25G>T	ABCB1	7	rs2235015	0.225	1.813 (1.032–3.185)	0.0424	+ve
CYP2C9_-1188T>C(5'UTR)	CYP2C9	10	rs4918758	0.281	1.720 (1.025–2.885)	0.0439	+ve
CYP4F2_c.918+67G>A	CYP4F2	19	rs3093158	0.326	0.605 (0.374–0.978)	0.0441	−ve
HLA-DPB1:c.313A>G(Met105Val)	HLA-DPB1	6	rs1042151	0.225	1.687 (1.018–2.796)	0.04623	+ve
CYP2C9_52320G>C(3'UTR)	CYP2C9	10	rs1505	0.273	1.741 (1.010–3.001)	0.04987	+ve

Abbreviations: Chr.: chromosome number; DOI: direction of impact; MAF: minor allele frequency.

Supplementary Table 4. Top PGx markers associated with total active moiety plasma levels at a minimum P < 0.05

Marker name	Associated gene	Chr.	rsID	MAF	OR (95% CI)	P value	DOI
HLA-DRA(rs9268542)	HLA-DRA	6	rs9268542	0.489	1.213 (1.047–1.405)	0.01284	+ve
UGT2B17_c.489G>A(E163=)	UGT2B17	4	rs34664906	0.344	1.209 (1.041–1.404)	0.01608	+ve
ABCB11_c.*236G>A(3'UTR)	ABCB11	2	rs473351	0.337	0.804 (0.674–0.959)	0.01863	−ve
UGT1A8_c.855+9429A>G	UGT1A8	2	rs1377460	0.0899	1.454 (1.071–1.975)	0.01973	+ve
ABCC3_c.-260T>A(5'UTR)	ABCC3	17	rs9895420	0.112	1.360 (1.054–1.754)	0.02123	+ve
HLA-DRA(rs9268528)	HLA-DRA	6	rs9268528	0.489	1.189 (1.029–1.375)	0.02272	+ve
UGT2B15_c.*185A>T(3'UTR)	UGT2B15	4	rs4148271	0.0899	0.732 (0.562–0.952)	0.02360	−ve
HLA-C(rs9461684)	HLA-C	6	rs9461684	0.124	0.706 (0.526–0.948)	0.02393	−ve
ABCG1_c.*399G>A(3'UTR)	ABCG1	21	rs1044317	0.416	1.184 (1.026–1.366)	0.02480	+ve
CYP2S1_1324C>G(P74=)	CYP2S1	19	rs338599	0.0506	1.448 (1.051–1.996)	0.02745	+ve
DPYD_c.1906-28506C>G	DPYD	1	rs4492658	0.421	0.820 (0.688–0.977)	0.03053	−ve
TYMS:c.*447_*452delTTAAAG	TYMS	18	rs151264360	0.399	1.207 (1.021–1.428)	0.03190	+ve
ABCB11_c.3084A>G(A1028=)	ABCB11	2	rs497692	0.421	0.821 (0.688–0.980)	0.03346	−ve
ABCB11_c.*368G>A(3'UTR)	ABCB11	2	rs495714	0.421	0.821 (0.688–0.980)	0.03346	−ve
ABCB11_c.*420A>G(3'UTR)	ABCB11	2	rs496550	0.415	0.743 (0.638–0.865)	0.03346	−ve
GSTP1*C_c.341C>T(A114V)	GSTP1	11	rs1138272	0.146	0.773 (0.612–0.976)	0.03429	−ve
DPYD_c.851-18271A>G	DPYD	1	rs2811196	0.23	1.190 (1.012–1.398)	0.03915	+ve
SLC22A1_c.156T>C(S52=)	SLC22A1	6	rs1867351	0.146	1.289 (1.017–1.634)	0.04052	+ve
DPYD_c.1906-5426A>G	DPYD	1	rs2152878	0.21	1.221 (1.012–1.473)	0.04166	+ve

Abbreviations: Chr.: chromosome number; DOI: direction of impact; MAF: minor allele frequency.

Supplementary Table 5. Top PGx markers associated with the RIS/9-OH-RIS metabolic ratio at a minimum $P < 0.05$

Marker name	Associated gene	Chr.	rsID	MAF	OR (95% CI)	P value	DOI
HLA-DRA(rs9268542)	HLA-DRA	6	rs9268542	0.489	1.213 (1.047–1.405)	0.01284	+ve
UGT2B17_c.489G>A(E163=)	UGT2B17	4	rs34664906	0.344	1.209 (1.041–1.404)	0.01608	+ve
ABCB11_c.*236G>A(3'UTR)	ABCB11	2	rs473351	0.337	0.804 (0.674–0.959)	0.01863	−ve
UGT1A8_c.855+9429A>G	UGT1A8	2	rs1377460	0.0899	1.454 (1.071–1.975)	0.01973	+ve
ABCC3_c.-260T>A(5'UTR)	ABCC3	17	rs9895420	0.112	1.360 (1.054–1.754)	0.02123	+ve
HLA-DRA(rs9268528)	HLA-DRA	6	rs9268528	0.489	1.189 (1.029–1.375)	0.02272	+ve
UGT2B15_c.*185A>T(3'UTR)	UGT2B15	4	rs4148271	0.0899	0.732 (0.562–0.952)	0.02360	−ve
HLA-C(rs9461684)	HLA-C	6	rs9461684	0.124	0.706 (0.526–0.948)	0.02393	−ve
ABCG1_c.*399G>A(3'UTR)	ABCG1	21	rs1044317	0.416	1.184 (1.026–1.366)	0.02480	+ve
CYP2S1_1324C>G(P74=)	CYP2S1	19	rs338599	0.0506	1.448 (1.051–1.996)	0.02745	+ve
DPYD_c.1906-28506C>G	DPYD	1	rs4492658	0.421	0.820 (0.688–0.977)	0.03053	−ve
TYMS:c.*447_*452delTTAAAG	TYMS	18	rs151264360	0.399	1.207 (1.021–1.428)	0.03190	+ve
ABCB11_c.3084A>G(A1028=)	ABCB11	2	rs497692	0.421	0.821 (0.688–0.980)	0.03346	−ve
ABCB11_c.*368G>A(3'UTR)	ABCB11	2	rs495714	0.421	0.821 (0.688–0.980)	0.03346	−ve
ABCB11_c.*420A>G(3'UTR)	ABCB11	2	rs496550	0.415	0.743 (0.638–0.865)	0.03346	−ve
GSTP1*C_c.341C>T(A114V)	GSTP1	11	rs1138272	0.146	0.773 (0.612–0.976)	0.03429	−ve
DPYD_c.851-18271A>G	DPYD	1	rs2811196	0.23	1.190 (1.012–1.398)	0.03915	+ve
SLC22A1_c.156T>C(S52=)	SLC22A1	6	rs1867351	0.146	1.289 (1.017–1.634)	0.04052	+ve
DPYD_c.1906-5426A>G	DPYD	1	rs2152878	0.21	1.221 (1.012–1.473)	0.04166	+ve

Abbreviations: Chr.: chromosome number; DOI: direction of impact; MAF: minor allele frequency.
