# Supplementary figures and images for: Exploratory focused pharmacogenetic testing reveals novel markers associated with risperidone pharmacokinetics in Saudi children with autism
Source: Front Pharmacol. 2024 Feb 5;15:1356763. doi: 10.3389/fphar.2024.1356763 (PMC10875102; doi:10.3389/fphar.2024.1356763)

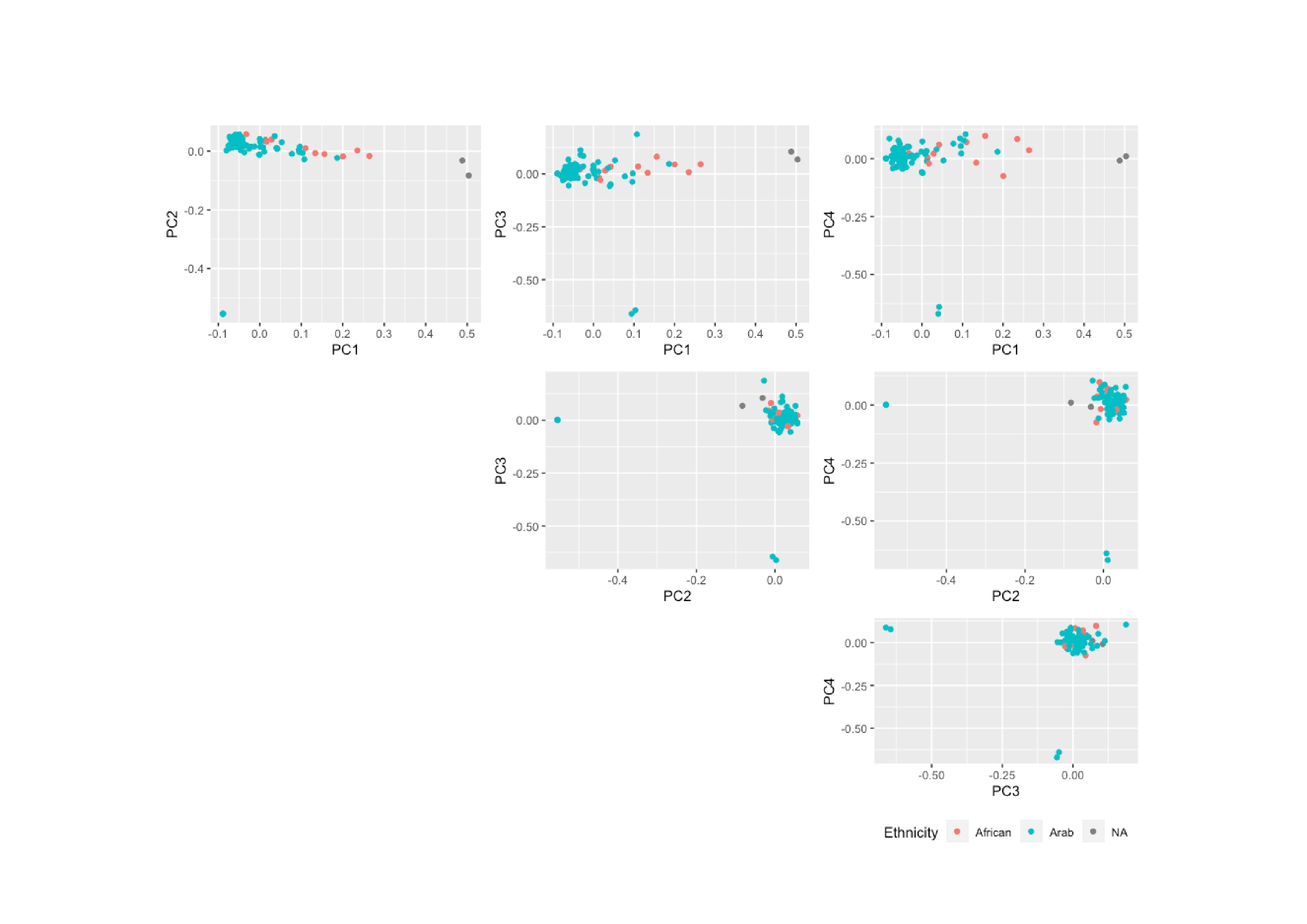

Supplement: Supplementary file 2 [file Image1.jpg]
